# Supplementary material for: Content-rich biological network constructed by mining PubMed abstracts
Source: BMC Bioinformatics. 2004 Oct 8;5:147. doi: 10.1186/1471-2105-5-147 (PMC528731; doi:10.1186/1471-2105-5-147)
Supplement: Additional File 2 — The original results of the above study (non-essential files are deleted to keep the file size under the limit set by BMC bioinformatics). [file 1471-2105-5-147-S2.bz2 › chilibotAdditionalFile2/dip05/114ID8645696E458/html/ATPD_ATPH.html]

 


 **ATPD** and **ATPH** 
  
Found 5 abstracts in PubMed, retrieved 05.  
 

 What does Google say? 
 PDF only 
| .edu only 

---

**Interactive relationship** (e.g. stimulation, inhibition, etc)

**Non-interactive relationship** (e.g. studied together, co-existance, homology, etc.)

- This cluster, denoted atp1, consists of four F0 genes and three F1 genes encoding the subunits a atpI, c  **atpH** , b atpG, b atpF, delta  **atpD** , alpha aptA, and gamma atpC in that order.  Ref: 2900236 J Bacteriol, 1988
- These fragments contain six closely linked reading frames that were identified as atpI,  **atpH** , atpG, atpF,  **atpD** , and atpA, coding for subunits IV, III, II, I, delta, and alpha, respectively.  Ref: 1532839 J Mol Biol, 1992
- The C. thermoaceticum atp operon contained nine genes arranged in the order atpI i, atpB a, atpE c, atpF b,  **atpH**  delta, atpA alpha, atpG gamma,  **atpD**  beta, and atpC epsilon.  Ref: 9171425 J Bacteriol, 1997
- Eight genes atpI, atpB, atpE 1, atpE 2, atpE 3, atpF,  **atpH** , and atpA upstream of and contiguous with the previously described genes atpG,  **atpD** , and atpC were cloned from chromosomal DNA of Acetobacterium woodii.  Ref: 10567365 J Biol Chem, 1999
